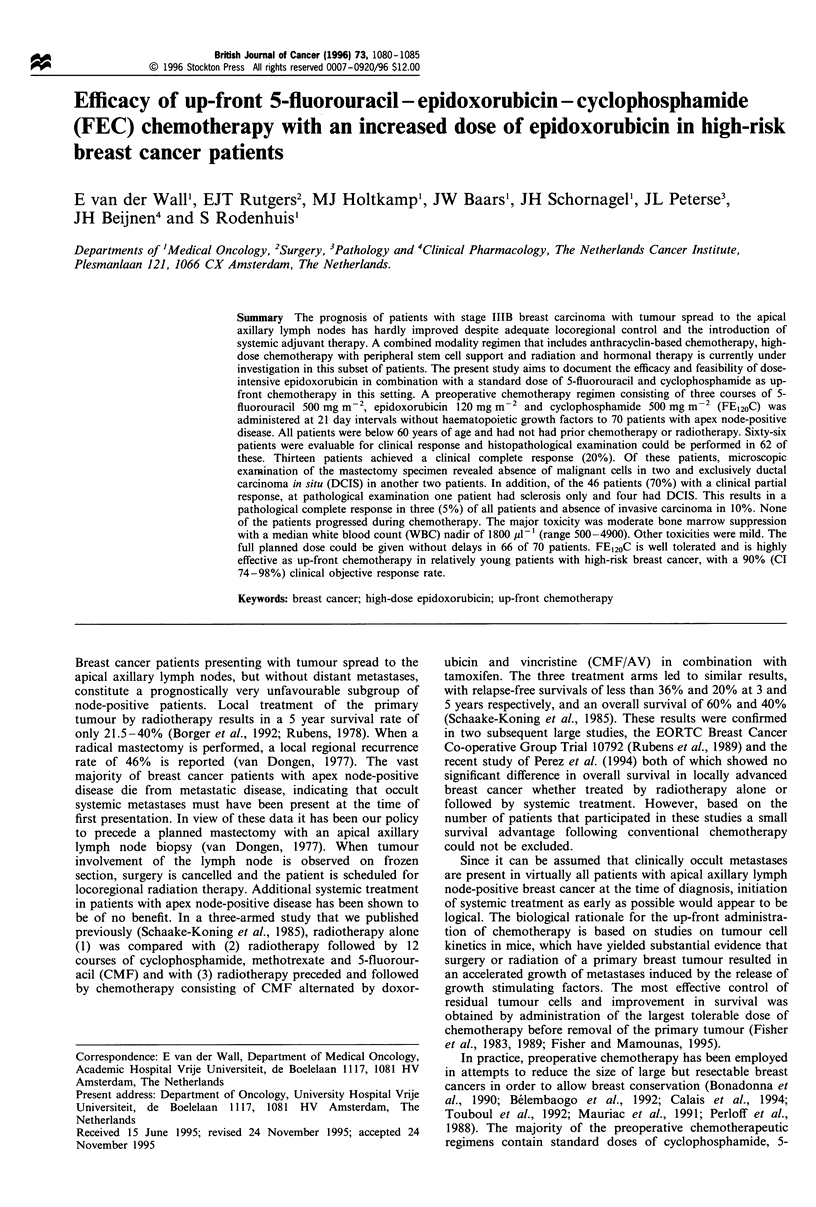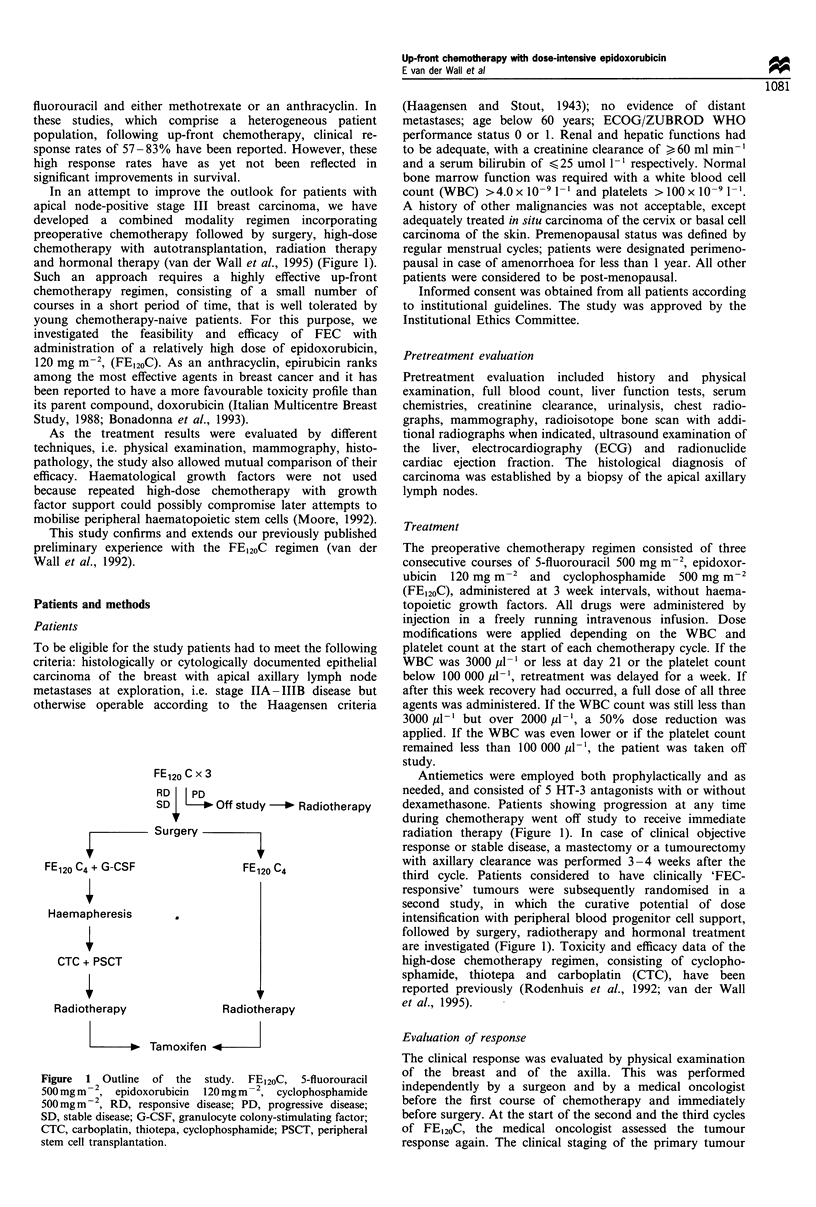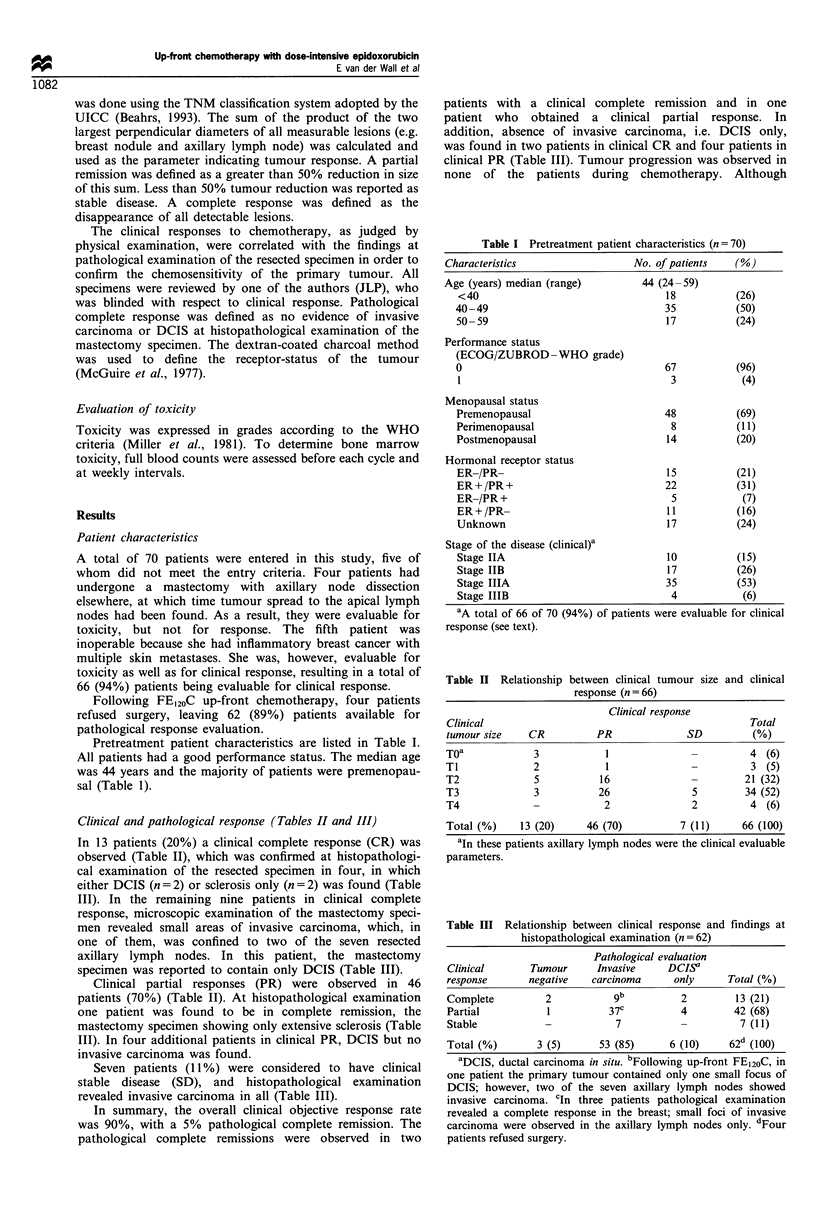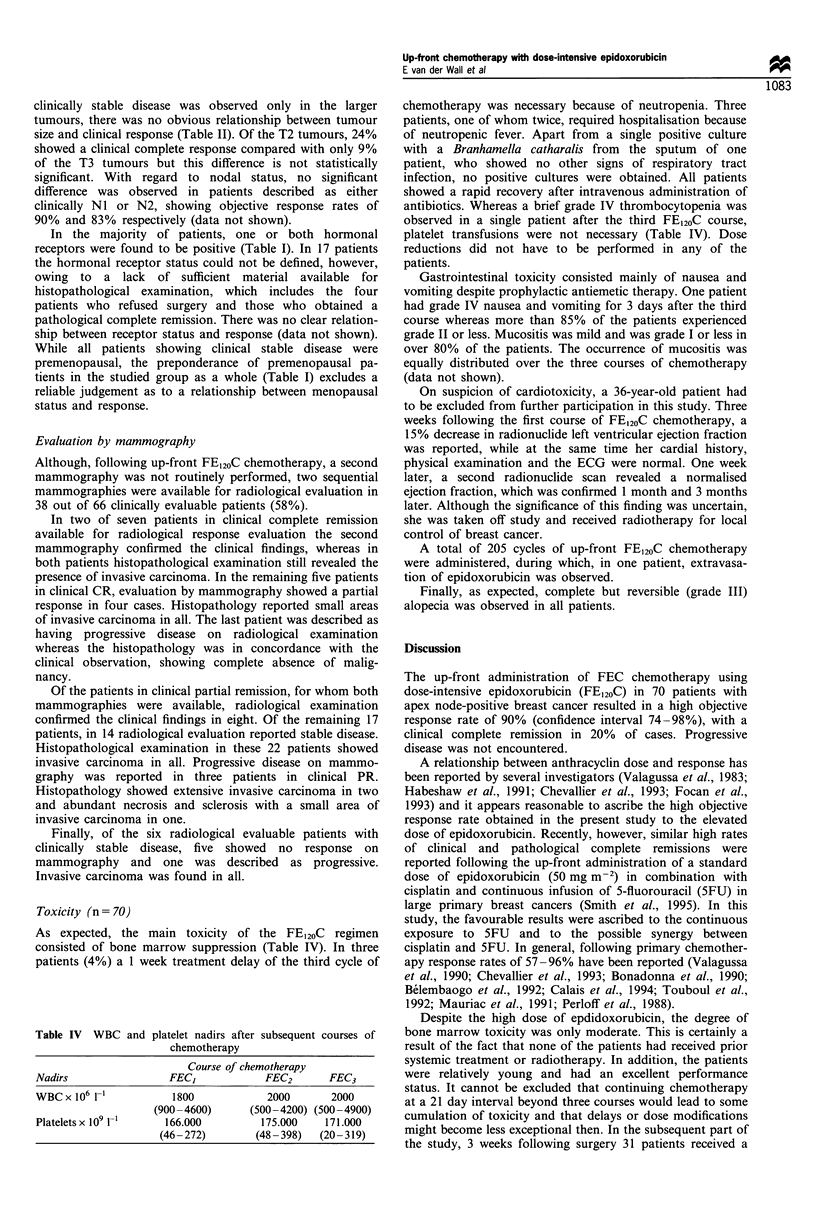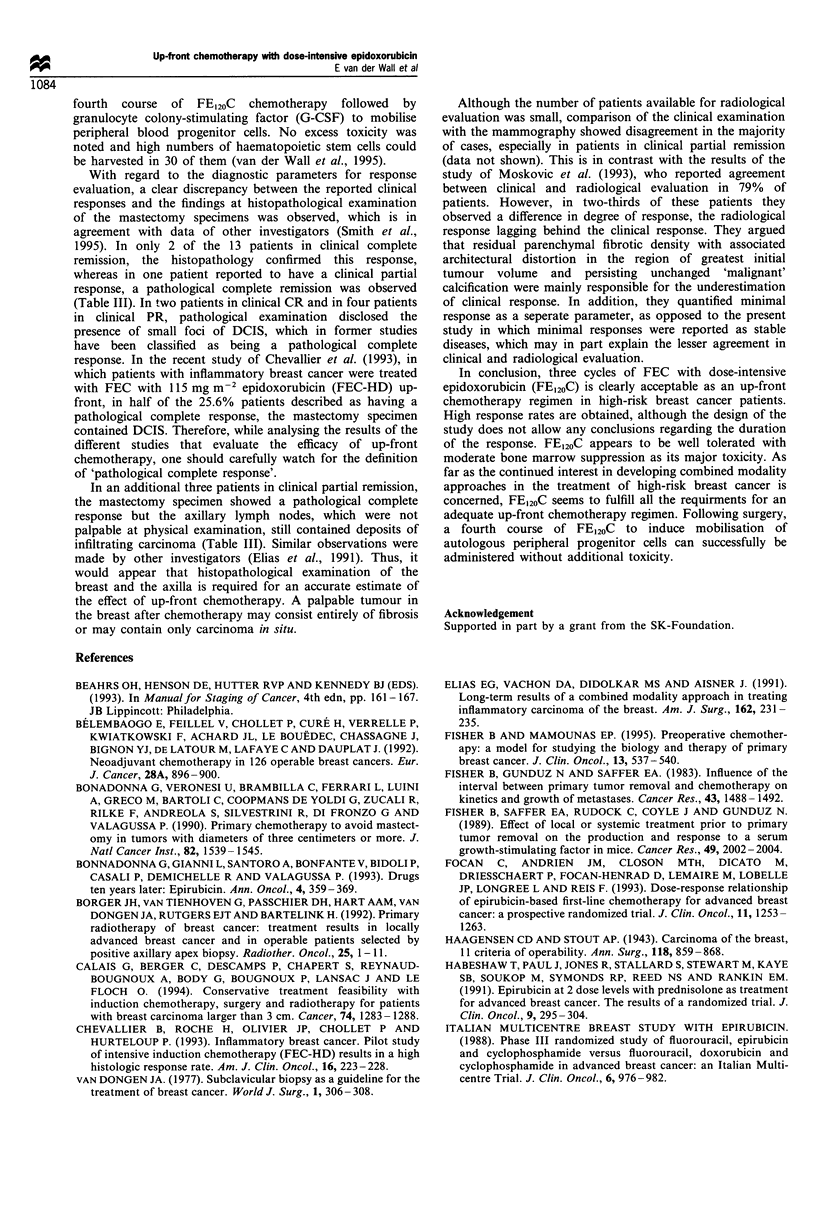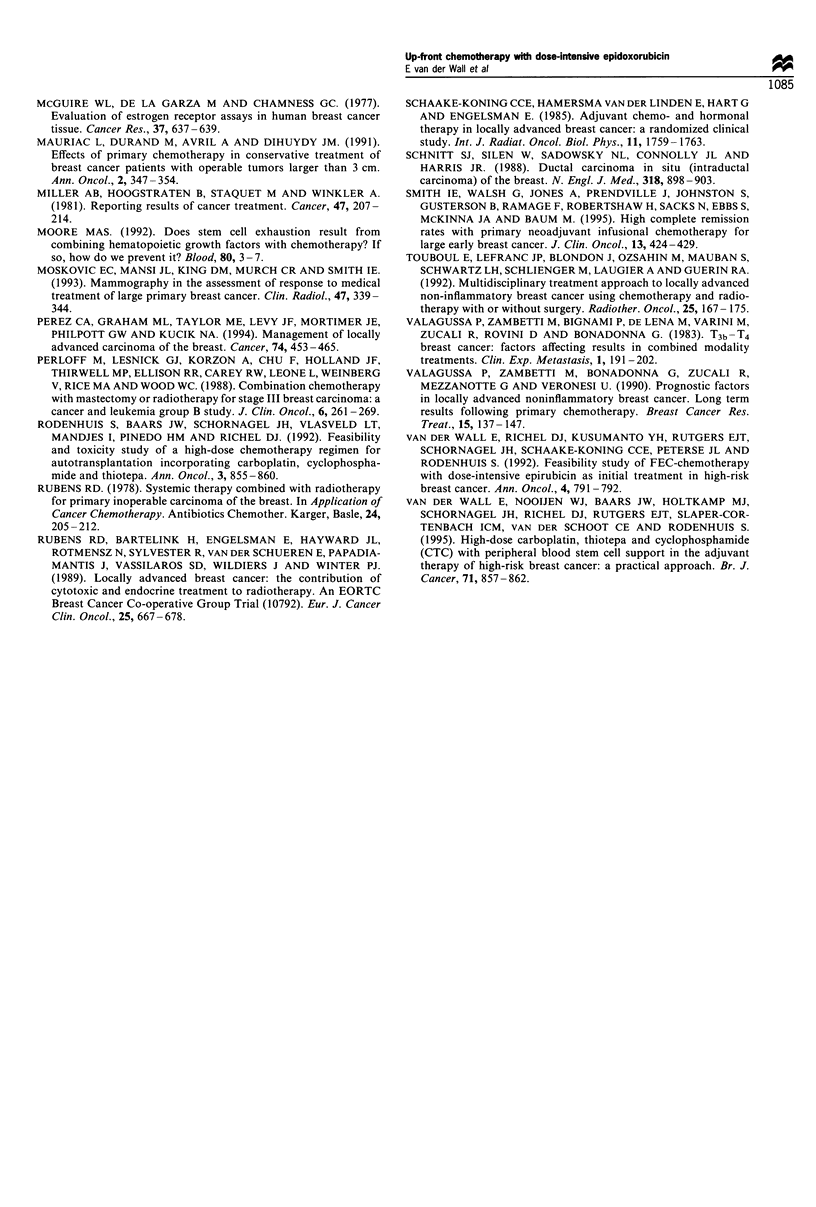# Efficacy of up-front 5-fluorouracil-epidoxorubicin-cyclophosphamide (FEC) chemotherapy with an increased dose of epidoxorubicin in high-risk breast cancer patients.

**DOI:** 10.1038/bjc.1996.208

**Published:** 1996-05

**Authors:** E. van der Wall, E. J. Rutgers, M. J. Holtkamp, J. W. Baars, J. H. Schornagel, J. L. Peterse, J. H. Beijnen, S. Rodenhuis

**Affiliations:** Department of Medical Oncology, The Netherlands Cancer Institute, Amsterdam, The Netherlands.

## Abstract

The prognosis of patients with stage IIIB breast carcinoma with tumour spread to the apical axillary lymph nodes has hardly improved despite adequate locoregional control and the introduction of systemic adjuvant therapy. A combined modality regimen that includes anthracyclin-based chemotherapy, high-dose chemotherapy with peripheral stem cell support and radiation and hormonal therapy is currently under investigation in this subset of patients. The present study aims to document the efficacy and feasibility of dose-intensive epidoxorubicin in combination with a standard dose of 5-fluorouracil and cyclophosphamide as up-front chemotherapy in this setting. A preoperative chemotherapy regimen consisting of three courses of 5-fluorouracil 500 mg m-2, epidoxorubicin 120 mg m-2 and cyclophosphamide 500 mg m-2 (FE120C) was administered at 21 day intervals without haematopoietic growth factors to 70 patients with apex node-positive disease. All patients were below 60 years of age and had not had prior chemotherapy or radiotherapy. Sixty-six patients were evaluable for clinical response and histopathological examination could be performed in 62 of these. Thirteen patients achieved a clinical complete response (20%). Of these patients, microscopic examination of the mastectomy specimen revealed absence of malignant cells in two and exclusively ductal carcinoma in situ (DCIS) in another two patients. In addition, of the 46 patients (70%) with a clinical partial response, at pathological examination one patient had sclerosis only and four had DCIS. This results in a pathological complete response in three (5%) of all patients and absence of invasive carcinoma in 10%. None of the patients progressed during chemotherapy. The major toxicity was moderate bone marrow suppression with a median white blood count (WBC) nadir of 1800 microliters-1 (range 500-4900). Other toxicities were mild. The full planned dose could be given without delays in 66 of 70 patients FE120C is well tolerated and is highly effective as up-front chemotherapy in relatively young patients with high-risk breast cancer, with a 90% (CI 74-98%) clinical objective response rate.


British Journal of Cancer (1996) 73, 1080-1085
© 1996 Stockton Press All rights reserved 0007-0920/96 $12.00

### Efficacy of up-front 5-fluorouracil-epidoxorubicin-cyclophosphamide (FEC) chemotherapy with an increased dose of epidoxorubicin in high-risk breast cancer patients

E van der Wall[1], EJT Rutgers[2], MJ Holtkamp[1], JW Baars[1], JH Schornagel[1], JL Peterse[3], JH Beijnen[4] and S Rodenhuis[1]

*Departments of [1]Medical Oncology, [2]Surgery, [3]Pathology and [4]Clinical Pharmacology, The Netherlands Cancer Institute, Plesmanlaan 121, 1066 CX Amsterdam, The Netherlands.*

**Summary** The prognosis of patients with stage IIIB breast carcinoma with tumour spread to the apical axillary lymph nodes has hardly improved despite adequate locoregional control and the introduction of systemic adjuvant therapy. A combined modality regimen that includes anthracyclin-based chemotherapy, high-dose chemotherapy with peripheral stem cell support and radiation and hormonal therapy is currently under investigation in this subset of patients. The present study aims to document the efficacy and feasibility of dose-intensive epidoxorubicin in combination with a standard dose of 5-fluorouracil and cyclophosphamide as up-front chemotherapy in this setting. A preoperative chemotherapy regimen consisting of three courses of 5-fluorouracil 500 mg $m^{-2}$, epidoxorubicin 120 mg $m^{-2}$ and cyclophosphamide 500 mg $m^{-2}$ ($FE_{120}C$) was administered at 21 day intervals without haematopoietic growth factors to 70 patients with apex node-positive disease. All patients were below 60 years of age and had not had prior chemotherapy or radiotherapy. Sixty-six patients were evaluable for clinical response and histopathological examination could be performed in 62 of these. Thirteen patients achieved a clinical complete response (20%). Of these patients, microscopic examination of the mastectomy specimen revealed absence of malignant cells in two and exclusively ductal carcinoma *in situ* (DCIS) in another two patients. In addition, of the 46 patients (70%) with a clinical partial response, at pathological examination one patient had sclerosis only and four had DCIS. This results in a pathological complete response in three (5%) of all patients and absence of invasive carcinoma in 10%. None of the patients progressed during chemotherapy. The major toxicity was moderate bone marrow suppression with a median white blood count (WBC) nadir of 1800 $\mu l^{-1}$ (range 500-4900). Other toxicities were mild. The full planned dose could be given without delays in 66 of 70 patients. $FE_{120}C$ is well tolerated and is highly effective as up-front chemotherapy in relatively young patients with high-risk breast cancer, with a 90% (CI 74-98%) clinical objective response rate.

**Keywords:** breast cancer; high-dose epidoxorubicin; up-front chemotherapy

Breast cancer patients presenting with tumour spread to the apical axillary lymph nodes, but without distant metastases, constitute a prognostically very unfavourable subgroup of node-positive patients. Local treatment of the primary tumour by radiotherapy results in a 5 year survival rate of only 21.5-40% (Borger *et al.*, 1992; Rubens, 1978). When a radical mastectomy is performed, a local regional recurrence rate of 46% is reported (van Dongen, 1977). The vast majority of breast cancer patients with apex node-positive disease die from metastatic disease, indicating that occult systemic metastases must have been present at the time of first presentation. In view of these data it has been our policy to precede a planned mastectomy with an apical axillary lymph node biopsy (van Dongen, 1977). When tumour involvement of the lymph node is observed on frozen section, surgery is cancelled and the patient is scheduled for locoregional radiation therapy. Additional systemic treatment in patients with apex node-positive disease has been shown to be of no benefit. In a three-armed study that we published previously (Schaake-Koning *et al.*, 1985), radiotherapy alone (1) was compared with (2) radiotherapy followed by 12 courses of cyclophosphamide, methotrexate and 5-fluorouracil (CMF) and with (3) radiotherapy preceded and followed by chemotherapy consisting of CMF alternated by doxorubicin and vincristine (CMF/AV) in combination with tamoxifen. The three treatment arms led to similar results, with relapse-free survivals of less than 36% and 20% at 3 and 5 years respectively, and an overall survival of 60% and 40% (Schaake-Koning *et al.*, 1985). These results were confirmed in two subsequent large studies, the EORTC Breast Cancer Co-operative Group Trial 10792 (Rubens *et al.*, 1989) and the recent study of Perez *et al.* (1994) both of which showed no significant difference in overall survival in locally advanced breast cancer whether treated by radiotherapy alone or followed by systemic treatment. However, based on the number of patients that participated in these studies a small survival advantage following conventional chemotherapy could not be excluded.

Since it can be assumed that clinically occult metastases are present in virtually all patients with apical axillary lymph node-positive breast cancer at the time of diagnosis, initiation of systemic treatment as early as possible would appear to be logical. The biological rationale for the up-front administration of chemotherapy is based on studies on tumour cell kinetics in mice, which have yielded substantial evidence that surgery or radiation of a primary breast tumour resulted in an accelerated growth of metastases induced by the release of growth stimulating factors. The most effective control of residual tumour cells and improvement in survival was obtained by administration of the largest tolerable dose of chemotherapy before removal of the primary tumour (Fisher *et al.*, 1983, 1989; Fisher and Mamounas, 1995).

In practice, preoperative chemotherapy has been employed in attempts to reduce the size of large but resectable breast cancers in order to allow breast conservation (Bonadonna *et al.*, 1990; Bélembaogo *et al.*, 1992; Calais *et al.*, 1994; Touboul *et al.*, 1992; Mauriac *et al.*, 1991; Perloff *et al.*, 1988). The majority of the preoperative chemotherapeutic regimens contain standard doses of cyclophosphamide, 5-

Correspondence: E van der Wall, Department of Medical Oncology, Academic Hospital Vrije Universiteit, de Boelelaan 1117, 1081 HV Amsterdam, The Netherlands
Present address: Department of Oncology, University Hospital Vrije Universiteit, de Boelelaan 1117, 1081 HV Amsterdam, The Netherlands
Received 15 June 1995; revised 24 November 1995; accepted 24 November 1995

fluorouracil and either methotrexate or an anthracyclin. In these studies, which comprise a heterogeneous patient population, following up-front chemotherapy, clinical response rates of 57–83% have been reported. However, these high response rates have as yet not been reflected in significant improvements in survival.

In an attempt to improve the outlook for patients with apical node-positive stage III breast carcinoma, we have developed a combined modality regimen incorporating preoperative chemotherapy followed by surgery, high-dose chemotherapy with autotransplantation, radiation therapy and hormonal therapy (van der Wall *et al.*, 1995) (Figure 1). Such an approach requires a highly effective up-front chemotherapy regimen, consisting of a small number of courses in a short period of time, that is well tolerated by young chemotherapy-naive patients. For this purpose, we investigated the feasibility and efficacy of FEC with administration of a relatively high dose of epidoxorubicin, 120 mg $m^{-2}$, ($FE_{120}C$). As an anthracyclin, epirubicin ranks among the most effective agents in breast cancer and it has been reported to have a more favourable toxicity profile than its parent compound, doxorubicin (Italian Multicentre Breast Study, 1988; Bonadonna *et al.*, 1993).

As the treatment results were evaluated by different techniques, i.e. physical examination, mammography, histopathology, the study also allowed mutual comparison of their efficacy. Haematological growth factors were not used because repeated high-dose chemotherapy with growth factor support could possibly compromise later attempts to mobilise peripheral haematopoietic stem cells (Moore, 1992).

This study confirms and extends our previously published preliminary experience with the $FE_{120}C$ regimen (van der Wall *et al.*, 1992).

### Patients and methods

### *Patients*

To be eligible for the study patients had to meet the following criteria: histologically or cytologically documented epithelial carcinoma of the breast with apical axillary lymph node metastases at exploration, i.e. stage IIA–IIIB disease but otherwise operable according to the Haagensen criteria

**Figure 1** Outline of the study. $FE_{120}C$, 5-fluorouracil 500 mg $m^{-2}$, epidoxorubicin 120 mg $m^{-2}$, cyclophosphamide 500 mg $m^{-2}$, RD, responsive disease; PD, progressive disease; SD, stable disease; G-CSF, granulocyte colony-stimulating factor; CTC, carboplatin, thiotepa, cyclophosphamide; PSCT, peripheral stem cell transplantation.

(Haagensen and Stout, 1943); no evidence of distant metastases; age below 60 years; ECOG/ZUBROD WHO performance status 0 or 1. Renal and hepatic functions had to be adequate, with a creatinine clearance of $\geqslant 60$ ml $min^{-1}$ and a serum bilirubin of $\leqslant 25$ umol $l^{-1}$ respectively. Normal bone marrow function was required with a white blood cell count (WBC) $>4.0 \times 10^{-9}$ $l^{-1}$ and platelets $>100 \times 10^{-9}$ $l^{-1}$. A history of other malignancies was not acceptable, except adequately treated *in situ* carcinoma of the cervix or basal cell carcinoma of the skin. Premenopausal status was defined by regular menstrual cycles; patients were designated perimenopausal in case of amenorrhoea for less than 1 year. All other patients were considered to be post-menopausal.

Informed consent was obtained from all patients according to institutional guidelines. The study was approved by the Institutional Ethics Committee.

### *Pretreatment evaluation*

Pretreatment evaluation included history and physical examination, full blood count, liver function tests, serum chemistries, creatinine clearance, urinalysis, chest radiographs, mammography, radioisotope bone scan with additional radiographs when indicated, ultrasound examination of the liver, electrocardiography (ECG) and radionuclide cardiac ejection fraction. The histological diagnosis of carcinoma was established by a biopsy of the apical axillary lymph nodes.

### *Treatment*

The preoperative chemotherapy regimen consisted of three consecutive courses of 5-fluorouracil 500 mg $m^{-2}$, epidoxorubicin 120 mg $m^{-2}$ and cyclophosphamide 500 mg $m^{-2}$ ($FE_{120}C$), administered at 3 week intervals, without haematopoietic growth factors. All drugs were administered by injection in a freely running intravenous infusion. Dose modifications were applied depending on the WBC and platelet count at the start of each chemotherapy cycle. If the WBC was 3000 $\mu l^{-1}$ or less at day 21 or the platelet count below 100 000 $\mu l^{-1}$, retreatment was delayed for a week. If after this week recovery had occurred, a full dose of all three agents was administered. If the WBC count was still less than 3000 $\mu l^{-1}$ but over 2000 $\mu l^{-1}$, a 50% dose reduction was applied. If the WBC was even lower or if the platelet count remained less than 100 000 $\mu l^{-1}$, the patient was taken off study.

Antiemetics were employed both prophylactically and as needed, and consisted of 5 HT-3 antagonists with or without dexamethasone. Patients showing progression at any time during chemotherapy went off study to receive immediate radiation therapy (Figure 1). In case of clinical objective response or stable disease, a mastectomy or a tumourectomy with axillary clearance was performed 3–4 weeks after the third cycle. Patients considered to have clinically 'FEC-responsive' tumours were subsequently randomised in a second study, in which the curative potential of dose intensification with peripheral blood progenitor cell support, followed by surgery, radiotherapy and hormonal treatment are investigated (Figure 1). Toxicity and efficacy data of the high-dose chemotherapy regimen, consisting of cyclophosphamide, thiotepa and carboplatin (CTC), have been reported previously (Rodenhuis *et al.*, 1992; van der Wall *et al.*, 1995).

### *Evaluation of response*

The clinical response was evaluated by physical examination of the breast and of the axilla. This was performed independently by a surgeon and by a medical oncologist before the first course of chemotherapy and immediately before surgery. At the start of the second and the third cycles of $FE_{120}C$, the medical oncologist assessed the tumour response again. The clinical staging of the primary tumour was done using the TNM classification system adopted by the UICC (Beahrs, 1993). The sum of the product of the two largest perpendicular diameters of all measurable lesions (e.g. breast nodule and axillary lymph node) was calculated and used as the parameter indicating tumour response. A partial remission was defined as a greater than 50% reduction in size of this sum. Less than 50% tumour reduction was reported as stable disease. A complete response was defined as the disappearance of all detectable lesions.

The clinical responses to chemotherapy, as judged by physical examination, were correlated with the findings at pathological examination of the resected specimen in order to confirm the chemosensitivity of the primary tumour. All specimens were reviewed by one of the authors (JLP), who was blinded with respect to clinical response. Pathological complete response was defined as no evidence of invasive carcinoma or DCIS at histopathological examination of the mastectomy specimen. The dextran-coated charcoal method was used to define the receptor-status of the tumour (McGuire *et al.*, 1977).

### *Evaluation of toxicity*

Toxicity was expressed in grades according to the WHO criteria (Miller *et al.*, 1981). To determine bone marrow toxicity, full blood counts were assessed before each cycle and at weekly intervals.

### Results

### *Patient characteristics*

A total of 70 patients were entered in this study, five of whom did not meet the entry criteria. Four patients had undergone a mastectomy with axillary node dissection elsewhere, at which time tumour spread to the apical lymph nodes had been found. As a result, they were evaluable for toxicity, but not for response. The fifth patient was inoperable because she had inflammatory breast cancer with multiple skin metastases. She was, however, evaluable for toxicity as well as for clinical response, resulting in a total of 66 (94%) patients being evaluable for clinical response.

Following $FE_{120}C$ up-front chemotherapy, four patients refused surgery, leaving 62 (89%) patients available for pathological response evaluation.

Pretreatment patient characteristics are listed in Table I. All patients had a good performance status. The median age was 44 years and the majority of patients were premenopausal (Table 1).

### *Clinical and pathological response (Tables II and III)*

In 13 patients (20%) a clinical complete response (CR) was observed (Table II), which was confirmed at histopathological examination of the resected specimen in four, in which either DCIS ($n=2$) or sclerosis only ($n=2$) was found (Table III). In the remaining nine patients in clinical complete response, microscopic examination of the mastectomy specimen revealed small areas of invasive carcinoma, which, in one of them, was confined to two of the seven resected axillary lymph nodes. In this patient, the mastectomy specimen was reported to contain only DCIS (Table III).

Clinical partial responses (PR) were observed in 46 patients (70%) (Table II). At histopathological examination one patient was found to be in complete remission, the mastectomy specimen showing only extensive sclerosis (Table III). In four additional patients in clinical PR, DCIS but no invasive carcinoma was found.

Seven patients (11%) were considered to have clinical stable disease (SD), and histopathological examination revealed invasive carcinoma in all (Table III).

In summary, the overall clinical objective response rate was 90%, with a 5% pathological complete remission. The pathological complete remissions were observed in two patients with a clinical complete remission and in one patient who obtained a clinical partial response. In addition, absence of invasive carcinoma, i.e. DCIS only, was found in two patients in clinical CR and four patients in clinical PR (Table III). Tumour progression was observed in none of the patients during chemotherapy. Although

**Table I** Pretreatment patient characteristics ($n=70$)

| *Characteristics* | *No. of patients* | *(%)* |
|---|---|---|
| Age (years) median (range) | 44 (24–59) | |
| <40 | 18 | (26) |
| 40–49 | 35 | (50) |
| 50–59 | 17 | (24) |
| Performance status | | |
| (ECOG/ZUBROD–WHO grade) | | |
| 0 | 67 | (96) |
| 1 | 3 | (4) |
| Menopausal status | | |
| Premenopausal | 48 | (69) |
| Perimenopausal | 8 | (11) |
| Postmenopausal | 14 | (20) |
| Hormonal receptor status | | |
| ER–/PR– | 15 | (21) |
| ER+/PR+ | 22 | (31) |
| ER–/PR+ | 5 | (7) |
| ER+/PR– | 11 | (16) |
| Unknown | 17 | (24) |
| Stage of the disease (clinical)[a] | | |
| Stage IIA | 10 | (15) |
| Stage IIB | 17 | (26) |
| Stage IIIA | 35 | (53) |
| Stage IIIB | 4 | (6) |

[a]A total of 66 of 70 (94%) of patients were evaluable for clinical response (see text).

**Table II** Relationship between clinical tumour size and clinical response ($n=66$)

| *Clinical tumour size* | *Clinical response* *CR* | *PR* | *SD* | *Total* (%) |
|---|---|---|---|---|
| T0[a] | 3 | 1 | – | 4 (6) |
| T1 | 2 | 1 | – | 3 (5) |
| T2 | 5 | 16 | – | 21 (32) |
| T3 | 3 | 26 | 5 | 34 (52) |
| T4 | – | 2 | 2 | 4 (6) |
| Total (%) | 13 (20) | 46 (70) | 7 (11) | 66 (100) |

[a]In these patients axillary lymph nodes were the clinical evaluable parameters.

**Table III** Relationship between clinical response and findings at histopathological examination ($n=62$)

| *Clinical response* | *Pathological evaluation* *Tumour negative* | *Invasive carcinoma* | *DCIS[a] only* | *Total* (%) |
|---|---|---|---|---|
| Complete | 2 | 9[b] | 2 | 13 (21) |
| Partial | 1 | 37[c] | 4 | 42 (68) |
| Stable | – | 7 | – | 7 (11) |
| Total (%) | 3 (5) | 53 (85) | 6 (10) | 62[d] (100) |

[a]DCIS, ductal carcinoma *in situ*. [b]Following up-front $FE_{120}C$, in one patient the primary tumour contained only one small focus of DCIS; however, two of the seven axillary lymph nodes showed invasive carcinoma. [c]In three patients pathological examination revealed a complete response in the breast; small foci of invasive carcinoma were observed in the axillary lymph nodes only. [d]Four patients refused surgery.

clinically stable disease was observed only in the larger tumours, there was no obvious relationship between tumour size and clinical response (Table II). Of the T2 tumours, 24% showed a clinical complete response compared with only 9% of the T3 tumours but this difference is not statistically significant. With regard to nodal status, no significant difference was observed in patients described as either clinically N1 or N2, showing objective response rates of 90% and 83% respectively (data not shown).

In the majority of patients, one or both hormonal receptors were found to be positive (Table I). In 17 patients the hormonal receptor status could not be defined, however, owing to a lack of sufficient material available for histopathological examination, which includes the four patients who refused surgery and those who obtained a pathological complete remission. There was no clear relationship between receptor status and response (data not shown). While all patients showing clinical stable disease were premenopausal, the preponderance of premenopausal patients in the studied group as a whole (Table I) excludes a reliable judgement as to a relationship between menopausal status and response.

*Evaluation by mammography*

Although, following up-front $FE_{120}C$ chemotherapy, a second mammography was not routinely performed, two sequential mammographies were available for radiological evaluation in 38 out of 66 clinically evaluable patients (58%).

In two of seven patients in clinical complete remission available for radiological response evaluation the second mammography confirmed the clinical findings, whereas in both patients histopathological examination still revealed the presence of invasive carcinoma. In the remaining five patients in clinical CR, evaluation by mammography showed a partial response in four cases. Histopathology reported small areas of invasive carcinoma in all. The last patient was described as having progressive disease on radiological examination whereas the histopathology was in concordance with the clinical observation, showing complete absence of malignancy.

Of the patients in clinical partial remission, for whom both mammographies were available, radiological examination confirmed the clinical findings in eight. Of the remaining 17 patients, in 14 radiological evaluation reported stable disease. Histopathological examination in these 22 patients showed invasive carcinoma in all. Progressive disease on mammography was reported in three patients in clinical PR. Histopathology showed extensive invasive carcinoma in two and abundant necrosis and sclerosis with a small area of invasive carcinoma in one.

Finally, of the six radiological evaluable patients with clinically stable disease, five showed no response on mammography and one was described as progressive. Invasive carcinoma was found in all.

*Toxicity (n = 70)*

As expected, the main toxicity of the $FE_{120}C$ regimen consisted of bone marrow suppression (Table IV). In three patients (4%) a 1 week treatment delay of the third cycle of chemotherapy was necessary because of neutropenia. Three patients, one of whom twice, required hospitalisation because of neutropenic fever. Apart from a single positive culture with a *Branhamella catharalis* from the sputum of one patient, who showed no other signs of respiratory tract infection, no positive cultures were obtained. All patients showed a rapid recovery after intravenous administration of antibiotics. Whereas a brief grade IV thrombocytopenia was observed in a single patient after the third $FE_{120}C$ course, platelet transfusions were not necessary (Table IV). Dose reductions did not have to be performed in any of the patients.

Gastrointestinal toxicity consisted mainly of nausea and vomiting despite prophylactic antiemetic therapy. One patient had grade IV nausea and vomiting for 3 days after the third course whereas more than 85% of the patients experienced grade II or less. Mucositis was mild and was grade I or less in over 80% of the patients. The occurrence of mucositis was equally distributed over the three courses of chemotherapy (data not shown).

On suspicion of cardiotoxicity, a 36-year-old patient had to be excluded from further participation in this study. Three weeks following the first course of $FE_{120}C$ chemotherapy, a 15% decrease in radionuclide left ventricular ejection fraction was reported, while at the same time her cardial history, physical examination and the ECG were normal. One week later, a second radionuclide scan revealed a normalised ejection fraction, which was confirmed 1 month and 3 months later. Although the significance of this finding was uncertain, she was taken off study and received radiotherapy for local control of breast cancer.

A total of 205 cycles of up-front $FE_{120}C$ chemotherapy were administered, during which, in one patient, extravasation of epidoxorubicin was observed.

Finally, as expected, complete but reversible (grade III) alopecia was observed in all patients.

**Table IV** WBC and platelet nadirs after subsequent courses of chemotherapy

| Nadirs | Course of chemotherapy $FEC_1$ | $FEC_2$ | $FEC_3$ |
|---|---|---|---|
| WBC $\times 10^6$ $l^{-1}$ | 1800 (900–4600) | 2000 (500–4200) | 2000 (500–4900) |
| Platelets $\times 10^9$ $l^{-1}$ | 166.000 (46–272) | 175.000 (48–398) | 171.000 (20–319) |

### Discussion

The up-front administration of FEC chemotherapy using dose-intensive epidoxorubicin ($FE_{120}C$) in 70 patients with apex node-positive breast cancer resulted in a high objective response rate of 90% (confidence interval 74–98%), with a clinical complete remission in 20% of cases. Progressive disease was not encountered.

A relationship between anthracyclin dose and response has been reported by several investigators (Valagussa *et al.*, 1983; Habeshaw *et al.*, 1991; Chevallier *et al.*, 1993; Focan *et al.*, 1993) and it appears reasonable to ascribe the high objective response rate obtained in the present study to the elevated dose of epidoxorubicin. Recently, however, similar high rates of clinical and pathological complete remissions were reported following the up-front administration of a standard dose of epidoxorubicin (50 mg $m^{-2}$) in combination with cisplatin and continuous infusion of 5-fluorouracil (5FU) in large primary breast cancers (Smith *et al.*, 1995). In this study, the favourable results were ascribed to the continuous exposure to 5FU and to the possible synergy between cisplatin and 5FU. In general, following primary chemotherapy response rates of 57–96% have been reported (Valagussa *et al.*, 1990; Chevallier *et al.*, 1993; Bonadonna *et al.*, 1990; Bélembaogo *et al.*, 1992; Calais *et al.*, 1994; Touboul *et al.*, 1992; Mauriac *et al.*, 1991; Perloff *et al.*, 1988).

Despite the high dose of epdidoxorubicin, the degree of bone marrow toxicity was only moderate. This is certainly a result of the fact that none of the patients had received prior systemic treatment or radiotherapy. In addition, the patients were relatively young and had an excellent performance status. It cannot be excluded that continuing chemotherapy at a 21 day interval beyond three courses would lead to some cumulation of toxicity and that delays or dose modifications might become less exceptional then. In the subsequent part of the study, 3 weeks following surgery 31 patients received a fourth course of $FE_{120}C$ chemotherapy followed by granulocyte colony-stimulating factor (G-CSF) to mobilise peripheral blood progenitor cells. No excess toxicity was noted and high numbers of haematopoietic stem cells could be harvested in 30 of them (van der Wall *et al.*, 1995).

With regard to the diagnostic parameters for response evaluation, a clear discrepancy between the reported clinical responses and the findings at histopathological examination of the mastectomy specimens was observed, which is in agreement with data of other investigators (Smith *et al.*, 1995). In only 2 of the 13 patients in clinical complete remission, the histopathology confirmed this response, whereas in one patient reported to have a clinical partial response, a pathological complete remission was observed (Table III). In two patients in clinical CR and in four patients in clinical PR, pathological examination disclosed the presence of small foci of DCIS, which in former studies have been classified as being a pathological complete response. In the recent study of Chevallier *et al.* (1993), in which patients with inflammatory breast cancer were treated with FEC with 115 mg $m^{-2}$ epidoxorubicin (FEC-HD) up-front, in half of the 25.6% patients described as having a pathological complete response, the mastectomy specimen contained DCIS. Therefore, while analysing the results of the different studies that evaluate the efficacy of up-front chemotherapy, one should carefully watch for the definition of 'pathological complete response'.

In an additional three patients in clinical partial remission, the mastectomy specimen showed a pathological complete response but the axillary lymph nodes, which were not palpable at physical examination, still contained deposits of infiltrating carcinoma (Table III). Similar observations were made by other investigators (Elias *et al.*, 1991). Thus, it would appear that histopathological examination of the breast and the axilla is required for an accurate estimate of the effect of up-front chemotherapy. A palpable tumour in the breast after chemotherapy may consist entirely of fibrosis or may contain only carcinoma *in situ*.

Although the number of patients available for radiological evaluation was small, comparison of the clinical examination with the mammography showed disagreement in the majority of cases, especially in patients in clinical partial remission (data not shown). This is in contrast with the results of the study of Moskovic *et al.* (1993), who reported agreement between clinical and radiological evaluation in 79% of patients. However, in two-thirds of these patients they observed a difference in degree of response, the radiological response lagging behind the clinical response. They argued that residual parenchymal fibrotic density with associated architectural distortion in the region of greatest initial tumour volume and persisting unchanged 'malignant' calcification were mainly responsible for the underestimation of clinical response. In addition, they quantified minimal response as a seperate parameter, as opposed to the present study in which minimal responses were reported as stable diseases, which may in part explain the lesser agreement in clinical and radiological evaluation.

In conclusion, three cycles of FEC with dose-intensive epidoxorubicin ($FE_{120}C$) is clearly acceptable as an up-front chemotherapy regimen in high-risk breast cancer patients. High response rates are obtained, although the design of the study does not allow any conclusions regarding the duration of the response. $FE_{120}C$ appears to be well tolerated with moderate bone marrow suppression as its major toxicity. As far as the continued interest in developing combined modality approaches in the treatment of high-risk breast cancer is concerned, $FE_{120}C$ seems to fulfill all the requirments for an adequate up-front chemotherapy regimen. Following surgery, a fourth course of $FE_{120}C$ to induce mobilisation of autologous peripheral progenitor cells can successfully be administered without additional toxicity.

**Acknowledgement**
Supported in part by a grant from the SK-Foundation.

### References

BEAHRS OH, HENSON DE, HUTTER RVP AND KENNEDY BJ (EDS). (1993). In *Manual for Staging of Cancer*, 4th edn, pp. 161–167. JB Lippincott: Philadelphia.

BÉLEMBAOGO E, FEILLEL V, CHOLLET P, CURÉ H, VERRELLE P, KWIATKOWSKI F, ACHARD JL, LE BOUËDEC, CHASSAGNE J, BIGNON YJ, DE LATOUR M, LAFAYE C AND DAUPLAT J. (1992). Neoadjuvant chemotherapy in 126 operable breast cancers. *Eur. J. Cancer*, **28A**, 896–900.

BONADONNA G, VERONESI U, BRAMBILLA C, FERRARI L, LUINI A, GRECO M, BARTOLI C, COOPMANS DE YOLDI G, ZUCALI R, RILKE F, ANDREOLA S, SILVESTRINI R, DI FRONZO G AND VALAGUSSA P. (1990). Primary chemotherapy to avoid mastectomy in tumors with diameters of three centimeters or more. *J. Natl Cancer Inst.*, **82**, 1539–1545.

BONNADONNA G, GIANNI L, SANTORO A, BONFANTE V, BIDOLI P, CASALI P, DEMICHELLE R AND VALAGUSSA P. (1993). Drugs ten years later: Epirubicin. *Ann. Oncol.*, **4**, 359–369.

BORGER JH, VAN TIENHOVEN G, PASSCHIER DH, HART AAM, VAN DONGEN JA, RUTGERS EJT AND BARTELINK H. (1992). Primary radiotherapy of breast cancer: treatment results in locally advanced breast cancer and in operable patients selected by positive axillary apex biopsy. *Radiother. Oncol.*, **25**, 1–11.

CALAIS G, BERGER C, DESCAMPS P, CHAPERT S, REYNAUD-BOUGNOUX A, BODY G, BOUGNOUX P, LANSAC J AND LE FLOCH O. (1994). Conservative treatment feasibility with induction chemotherapy, surgery and radiotherapy for patients with breast carcinoma larger than 3 cm. *Cancer*, **74**, 1283–1288.

CHEVALLIER B, ROCHE H, OLIVIER JP, CHOLLET P AND HURTELOUP P. (1993). Inflammatory breast cancer. Pilot study of intensive induction chemotherapy (FEC-HD) results in a high histologic response rate. *Am. J. Clin. Oncol.*, **16**, 223–228.

VAN DONGEN JA. (1977). Subclavicular biopsy as a guideline for the treatment of breast cancer. *World J. Surg.*, **1**, 306–308.

ELIAS EG, VACHON DA, DIDOLKAR MS AND AISNER J. (1991). Long-term results of a combined modality approach in treating inflammatory carcinoma of the breast. *Am. J. Surg.*, **162**, 231–235.

FISHER B AND MAMOUNAS EP. (1995). Preoperative chemotherapy: a model for studying the biology and therapy of primary breast cancer. *J. Clin. Oncol.*, **13**, 537–540.

FISHER B, GUNDUZ N AND SAFFER EA. (1983). Influence of the interval between primary tumor removal and chemotherapy on kinetics and growth of metastases. *Cancer Res.*, **43**, 1488–1492.

FISHER B, SAFFER EA, RUDOCK C, COYLE J AND GUNDUZ N. (1989). Effect of local or systemic treatment prior to primary tumor removal on the production and response to a serum growth-stimulating factor in mice. *Cancer Res.*, **49**, 2002–2004.

FOCAN C, ANDRIEN JM, CLOSON MTH, DICATO M, DRIESSCHAERT P, FOCAN-HENRAD D, LEMAIRE M, LOBELLE JP, LONGREE L AND REIS F. (1993). Dose-response relationship of epirubicin-based first-line chemotherapy for advanced breast cancer: a prospective randomized trial. *J. Clin. Oncol.*, **11**, 1253–1263.

HAAGENSEN CD AND STOUT AP. (1943). Carcinoma of the breast, 11 criteria of operability. *Ann. Surg.*, **118**, 859–868.

HABESHAW T, PAUL J, JONES R, STALLARD S, STEWART M, KAYE SB, SOUKOP M, SYMONDS RP, REED NS AND RANKIN EM. (1991). Epirubicin at 2 dose levels with prednisolone as treatment for advanced breast cancer. The results of a randomized trial. *J. Clin. Oncol.*, **9**, 295–304.

ITALIAN MULTICENTRE BREAST STUDY WITH EPIRUBICIN. (1988). Phase III randomized study of fluorouracil, epirubicin and cyclophosphamide versus fluorouracil, doxorubicin and cyclophosphamide in advanced breast cancer: an Italian Multicentre Trial. *J. Clin. Oncol.*, **6**, 976–982.

McGuire WL, De La Garza M and Chamness GC. (1977). Evaluation of estrogen receptor assays in human breast cancer tissue. *Cancer Res.*, **37**, 637–639.

Mauriac L, Durand M, Avril A and Dihuydy JM. (1991). Effects of primary chemotherapy in conservative treatment of breast cancer patients with operable tumors larger than 3 cm. *Ann. Oncol.*, **2**, 347–354.

Miller AB, Hoogstraten B, Staquet M and Winkler A. (1981). Reporting results of cancer treatment. *Cancer*, **47**, 207–214.

Moore MAS. (1992). Does stem cell exhaustion result from combining hematopoietic growth factors with chemotherapy? If so, how do we prevent it? *Blood*, **80**, 3–7.

Moskovic EC, Mansi JL, King DM, Murch CR and Smith IE. (1993). Mammography in the assessment of response to medical treatment of large primary breast cancer. *Clin. Radiol.*, **47**, 339–344.

Perez CA, Graham ML, Taylor ME, Levy JF, Mortimer JE, Philpott GW and Kucik NA. (1994). Management of locally advanced carcinoma of the breast. *Cancer*, **74**, 453–465.

Perloff M, Lesnick GJ, Korzon A, Chu F, Holland JF, Thirwell MP, Ellison RR, Carey RW, Leone L, Weinberg V, Rice MA and Wood WC. (1988). Combination chemotherapy with mastectomy or radiotherapy for stage III breast carcinoma: a cancer and leukemia group B study. *J. Clin. Oncol.*, **6**, 261–269.

Rodenhuis S, Baars JW, Schornagel JH, Vlasveld LT, Mandjes I, Pinedo HM and Richel DJ. (1992). Feasibility and toxicity study of a high-dose chemotherapy regimen for autotransplantation incorporating carboplatin, cyclophosphamide and thiotepa. *Ann. Oncol.*, **3**, 855–860.

Rubens RD. (1978). Systemic therapy combined with radiotherapy for primary inoperable carcinoma of the breast. In *Application of Cancer Chemotherapy*. Antibiotics Chemother. Karger, Basle, **24**, 205–212.

Rubens RD, Bartelink H, Engelsman E, Hayward JL, Rotmensz N, Sylvester R, van der Schueren E, Papadiamantis J, Vassilaros SD, Wildiers J and Winter PJ. (1989). Locally advanced breast cancer: the contribution of cytotoxic and endocrine treatment to radiotherapy. An EORTC Breast Cancer Co-operative Group Trial (10792). *Eur. J. Cancer Clin. Oncol.*, **25**, 667–678.

Schaake-Koning CCE, Hamersma van der Linden E, Hart G and Engelsman E. (1985). Adjuvant chemo- and hormonal therapy in locally advanced breast cancer: a randomized clinical study. *Int. J. Radiat. Oncol. Biol. Phys.*, **11**, 1759–1763.

Schnitt SJ, Silen W, Sadowsky NL, Connolly JL and Harris JR. (1988). Ductal carcinoma in situ (intraductal carcinoma) of the breast. *N. Engl. J. Med.*, **318**, 898–903.

Smith IE, Walsh G, Jones A, Prendville J, Johnston S, Gusterson B, Ramage F, Robertshaw H, Sacks N, Ebbs S, McKinna JA and Baum M. (1995). High complete remission rates with primary neoadjuvant infusional chemotherapy for large early breast cancer. *J. Clin. Oncol.*, **13**, 424–429.

Touboul E, Lefranc JP, Blondon J, Ozsahin M, Mauban S, Schwartz LH, Schlienger M, Laugier A and Guerin RA. (1992). Multidisciplinary treatment approach to locally advanced non-inflammatory breast cancer using chemotherapy and radiotherapy with or without surgery. *Radiother. Oncol.*, **25**, 167–175.

Valagussa P, Zambetti M, Bignami P, de Lena M, Varini M, Zucali R, Rovini D and Bonadonna G. (1983). $T_{3b}$–$T_4$ breast cancer: factors affecting results in combined modality treatments. *Clin. Exp. Metastasis*, **1**, 191–202.

Valagussa P, Zambetti M, Bonadonna G, Zucali R, Mezzanotte G and Veronesi U. (1990). Prognostic factors in locally advanced noninflammatory breast cancer. Long term results following primary chemotherapy. *Breast Cancer Res. Treat.*, **15**, 137–147.

van der Wall E, Richel DJ, Kusumanto YH, Rutgers EJT, Schornagel JH, Schaake-Koning CCE, Peterse JL and Rodenhuis S. (1992). Feasibility study of FEC-chemotherapy with dose-intensive epirubicin as initial treatment in high-risk breast cancer. *Ann. Oncol.*, **4**, 791–792.

van der Wall E, Nooijen WJ, Baars JW, Holtkamp MJ, Schornagel JH, Richel DJ, Rutgers EJT, Slaper-Cortenbach ICM, van der Schoot CE and Rodenhuis S. (1995). High-dose carboplatin, thiotepa and cyclophosphamide (CTC) with peripheral blood stem cell support in the adjuvant therapy of high-risk breast cancer: a practical approach. *Br. J. Cancer*, **71**, 857–862.